# Tumor-informed liquid biopsy detection of structural variants in high grade serous ovarian cancer

**DOI:** 10.18632/oncoscience.645

**Published:** 2026-03-05

**Authors:** Jian Li, Shiro Takamatsu, Allison L. Brodsky, Thomas Welte, Katherine Calzoncinth, Veena K. Vuttaradhi, Joseph Celestino, Barrett Lawson, R. Tyler Hillman

**Affiliations:** ^1^Department of Gynecologic Oncology and Reproductive Medicine, Division of Surgery, The University of Texas M.D. Anderson Cancer Center, Houston, Texas 77030, USA; ^2^Department of Pathology, Division of Pathology/Lab Medicine, The University of Texas MD Anderson Cancer Center, Houston, TX 77030, USA; ^3^Department of Obstetrics, Rebecca and John Moores Cancer Center, Gynecology and Reproductive Sciences, Division of Gynecologic Oncology, University of California San Diego, La Jolla, CA 92093, USA

**Keywords:** ovarian cancer, ctDNA, biomarker, liquid biopsy, structural variant

## Abstract

Background: High grade serous ovarian cancer (HGSOC) recurs frequently and commercial tests have emerged for tumor-informed, cell-free DNA (cfDNA)-based detection of minimal residual disease. These tests are based on somatic single nucleotide variants prevalent in many cancers and thus are not well matched to HGSOC, which is dominated by structural genomic rearrangements. The purpose of this study was to evaluate the feasibility of a structural-variant (SV)-informed, cfDNA-based method for detecting clonal and subclonal HGSOC disease burden.

Methods: A method was developed for detecting patient-specific SV breakpoints using digital droplet PCR (ddPCR) with custom tumor-informed primer/probe pairs. Test parameters were first estimated using synthetic cfDNA generated by ultrasonication of genomic DNA from ovarian cancer cell lines. The optimized workflow was implemented in which whole genome sequencing of multisite pre-treatment HGSOC biopsies performed and high confidence SVs were called by multiple published SV callers. Real-time PCR and ddPCR were used for assay development.

Results: Following the optimized workflow, tumor-specific SV breakpoint-spanning primers/probe sets of four HGSOC patients’ multisite biopsies were designed and validated by real-time PCR and ddPCR. Together with four HGSOCs, a total of 29 SVs breakpoints-spanning tumor-informed primers/probe sets were designed and validated in multisite biopsies. 15 validated tumor-specific SVs were selected for quantification in their corresponding liquid biopsies using the validated ddPCR, and 9 had measurements in liquid biopsies.

Conclusions: Our result shows the detection of SVs from pre-treatment cfDNA using tumor-informed breakpoints-spanning ddPCR is feasible and may enable a novel and sensitive method for monitoring on-treatment disease burden.

## INTRODUCTION

High grade serous ovarian cancer (HGSOC) is the most common histological type of ovarian cancer and is the leading cause of death from gynecologic cancers [1]. Most HGSOC responds to combined first-line treatment with cytoreductive surgery and perioperative platinum-based chemotherapy, yet most patients will experience relapse which is the major cause of mortality in HGSOC. One fundamental mechanism of cancer recurrence is the outgrowth of resistant subclones present at initial diagnosis following elimination of sensitive subclones by chemotherapy [2, 3]. Therefore, understanding the genomic composition of these resistant subclones may provide insights in the mechanisms for recurrence and promising targets for the development of novel treatment. However, after the achievement of a clinical or pathologic complete response, the volume of minimal residual disease (MRD) is usually not sufficient for conventional genomic analysis or even clinical detection in many cases.

The detection of tumor DNA from circulation has emerged as an alternative to the analysis of tumor biopsies for an expanding range of clinical and research applications. For example, the FDA has approved a cfDNA assay for the prognostic molecular stratification of non-small cell lung cancer patients by EGFR mutation status [4]. Signatera^™^ is a commercially available platform for MRD monitoring in cancers by detection of tumor-informed single nucleotides variants (SNVs) in ctDNA. Most recent study on the usage of Signatera^™^ on epithelial ovarian cancers showed that the ctDNA outperformed CA-125 in identifying patients at highest risk of recurrence [5]. PCR and sequencing-based assays have also been developed as platforms for the early detection of relapse in breast cancer [6] and it has also been shown that cfDNA correlates with clinical and radiographic measurement of disease burden [7]. The early discovery that tumor specific TP53 mutations are detectable in bodily fluids of HGSOC patients [8] led to the development of assays for detecting cfDNA TP53 mutations as a means of monitoring disease status [9]. Baseline intra-tumoral heterogeneity gives rise to diverse tumor subclones that may respond differently to selective pressure from chemotherapy or precision therapies. In a large study of patients with colorectal cancer, cfDNA detected subclonal mutations in the extracellular domain of EGFR that may have reflected the on-treatment emergence of resistant subclones [10]. Since TP53 mutations are truncal in nearly all cases of HGSOC, it is not yet known if cfDNA can be effectively applied to the detection of structural variants (SVs) in this disease.

HGSOC is characterized by genomic instability and frequent SVs defined by at least one “breakpoint” or rearrangement site [11]. The high frequency of SVs in this disease and their close association with fundamental oncogenic mechanisms such as homologous recombination deficiency make them an ideal mutational substrate for tracking HGSOC tumor disease burden. In addition, the unique characteristics of tumor SVs allow for the design of tumor-informed primer/probe pairs that span breakpoints, lending a high degree of specificity. The long-term goal of this research is to develop a platform to detect and monitor on-treatment clonal dynamics in HGSOC in order to predict treatment response and investigate mechanisms of treatment resistance. Here we report the successful detection of SVs from circulating cell-free DNA (cfDNA) to monitor on-treatment HGSOC response.

## RESULTS

### Workflow of detection of SVs in liquid biopsy optimization

The feasibility of SV-based detection from liquid biopsy samples was first evaluated using synthetic cfDNA of ovarian cancer cell lines Caov-3 and SKOV-3. In these experiments, primer/probe sets designed to target SVs in one cell line were evaluated in the other cell line, in order to estimate sensitivity and specificity. The synthetic cfDNA with a peak size of 180bp, mimicking actual cfDNA distribution, of both cell lines was generated by ultrasonication of genomic DNA. Published WGS data of Caov-3 [11] were used for SVs analysis. Consensus of two SV calling algorithms, LUMPY [12] and BRASS [13], identified simple deletion (DEL) SVs in Caov-3. A total of 21 DEL SVs were selected for breakpoint-spanning primers design with a targeted amplicon of approximately 150bp.

11 out of 21 pairs of breakpoints-spanning primers showed specific amplification in synthetic cfDNA of Caov-3, not SKOV-3. To acquire the accurate sequences of SV coordinates, required for custom TaqMan probes, the 11 PCR amplicons of Caov-3 SVs were subcloned and analyzed using Sanger sequencing. Nine custom TaqMan probes of Caov-3 SVs were designed. All nine SV breakpoints-spanning primers/probe sets were screened by real-time PCR, one pair was eliminated for further analysis due to high amplification activity in no template control reactions (NTCs).

The remaining 8 DEL SVs all showed low ΔCq values in synthetic cfDNA of Caov-3, with no amplification detected in synthetic cfDNA of SKOV-3 (Supplementary Figure 1A). The final step to establish the workflow was to measure the concentrations of these Caov-3 SVs using the validated primers/probe sets in ddPCR (digital-droplet PCR). As shown in Supplementary Figure 1B, the concentrations of all 8 DEL SVs were measured in synthetic cfDNA of Caov-3, with no measurement in control synthetic cfDNA made from SKOV-3 gDNA. This result indicates the high specificity of the designed primers/probe assays, and the application in cfDNA from patient samples.

**Figure 1:**
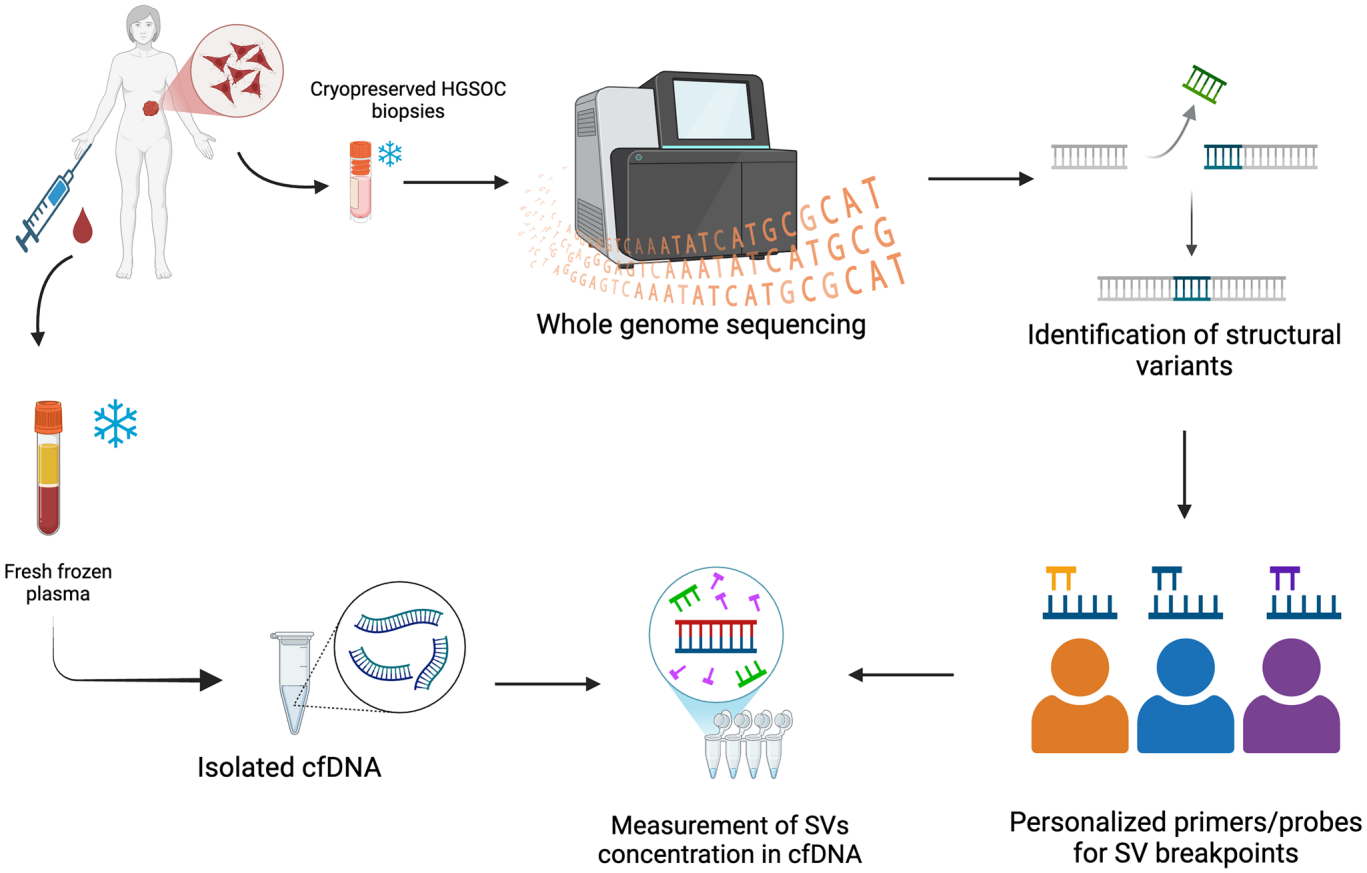
Workflow of detection of tumor-informed SVs of HGSOCs in liquid biopsy. Schematic summary of the workflow: cryopreserved HGSOC multisite biopsies and WBC (white blood cell, germline control) were subjected to WGS (whole genome sequencing), and SVs (structural variants) were identified by multiple algorithms; personalized primers/probes were designed and validated using gDNA (genomic DNA) of HGSOC biopsies before measuring their concentration in liquid biopsy; cfDNA was isolated from fresh frozen plasma.

Taken together, we streamlined the workflow (Figure 1) based on the testing results from ovarian cancer cell lines. We obtained pre-treatment cryopreserved multisite biopsy specimens and blood components of four patients who were diagnosed with HGSOC. Following SVs calling, SV breakpoints-spanning primers/probe design and validation using multisite biopsy samples, eventually selected high-specificity assays are used to measure the SVs concentrations in liquid biopsy.

### SVs identification in HGSOC multisite biopsies using NGS

**Figure 2:**
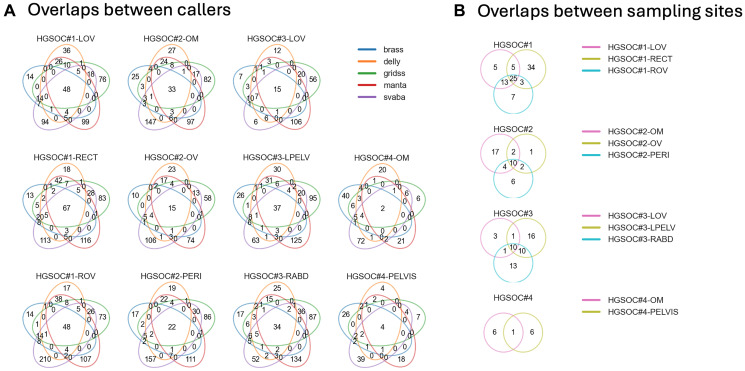
Identification and concordance of SVs across multisite biopsies using WGS. (**A**) Venn diagrams showing the overlap of SVs identified by five somatic SV callers at each sampling site. A substantial number of SVs were consistently detected by all five methods in HGSOC#1–#3, whereas HGSOC#4 showed a lower concordance, likely due to reduced sensitivity due to low tumor purity. (**B**) Venn diagrams showing the overlap of high-confidence SVs (see Methods) among different sampling sites within each case. Shared and unshared SVs reflect intratumor spatial heterogeneity.

We next performed WGS on 11 multisite biopsy samples with matched blood controls collected from four HGSOC cases. To minimize false positives and detect high-confidence SVs, we used five somatic SV callers and retained variants identified by at least four of them (Figure 2A, see Methods). While some of these high-confidence SVs were shared across multiple sites within the same patient, others were not (Figure 2B), indicating intratumor spatial heterogeneity of these cases.

### Validation of SVs breakpoints-spanning primers/probe sets by real-time PCR

After tumor-informed SVs from four HGSOC patients’ multisite biopsies were identified from WGS results, we selected DELs (deletions) of SVs for further investigation. Selection of SVs for primers/probe design was mainly based on the visualization of sequencing depth on Samplots (Supplementary Figure 2). Relative expressions of SVs to GAPDH were used for comparison. ΔCq was calculated as follows: ΔCq = Cq.Target - Cq.GAPDH. The lower ΔCq values, the higher relative expression of an SV target. For this study, ΔCq <5 is considered high expression, and >10 is an indication of low expression.

**Figure 3:**
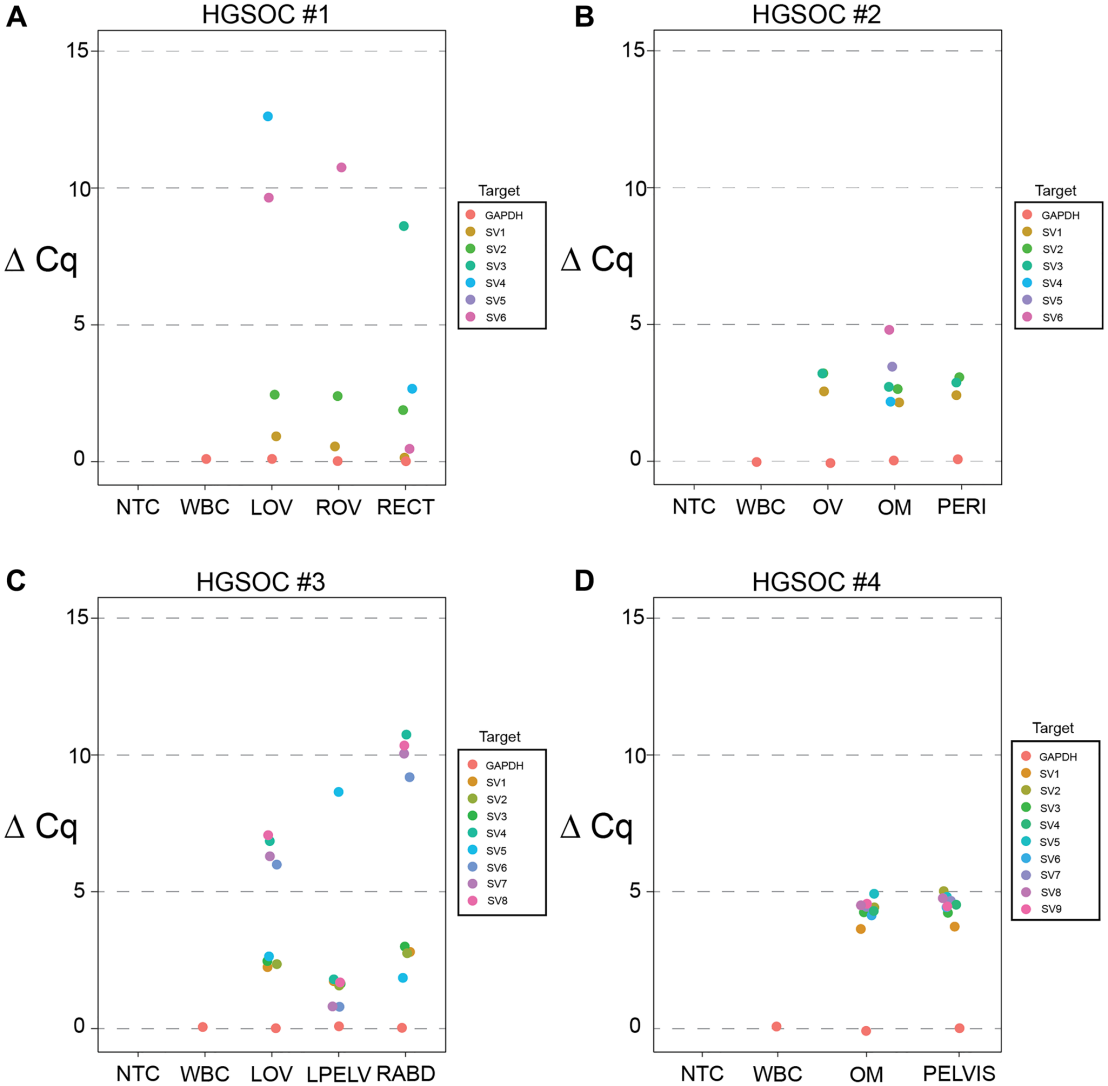
Validation of SVs breakpoints-spanning primers/probe sets by real-time PCR. (**A**–**D**) Real-time PCR results of targeted SVs. ΔCq (ΔCq = Cq.Target - Cq.GAPDH) were calculated and compared in HGSOC#1-4. Each dot represents the mean value of three duplicates. Germline control WBC (white blood cell) of each HGSOC and NTC (no template control) were used for all experiments. (A) In HGSOC#1, gDNA of multisite biopsies LOV (left ovary), ROV (right ovary), and RECT (rectus) were used for real-time PCR and 6 SV targets (SV1-6) were tested. (B) In HGSOC#2, gDNA of multisite biopsies OV (ovary), OM (omentum), and PERI (pericardium) were used for real-time PCR and 6 SV targets (SV1-6) were tested. (C) In HGSOC#3, gDNA of multisite biopsies LOV (left ovary), LPELV (left pelvis), and RABD (right abdominum) were used for real-time PCR and 8 SV targets (SV1-8) were tested. (D) In HGSOC#4, gDNA of biopsies OM (omentum) and PELVIS (pelvis) were used for real-time PCR and 9 SV targets (SV1-9) were tested.

In HGSOC#1 case, real-time PCR validated six SVs breakpoints-spanning primers/probe sets in LOV, ROV, and RECT biopsies (Figure 3A), among which, SV1 and SV2 showed relatively higher expression across all samples; SV3 had lower expression in the RECT biopsy site; SV4 had higher expression in the RECT site, little or no detection in the other sites; SV5 showed no detection in any biopsies; SV6 had higher expression in RECT and little in the rest sites. In HGSOC#2 case, real-time PCR validated six SVs breakpoints-spanning primers/probe sets in OV, OM, and PERI biopsies (Figure 3B). SV1 and SV3 showed higher levels in all biopsies; SV2 had been detected in both OM and PERI; SV4, SV5, and SV6 had specific expression only in OM.

In HGSOC#3, eight SVs breakpoints-spanning primers/probe sets were tested in LOV, LPELV, and RABD biopsies (Figure 3C) by real-time PCR. All eight SVs were detected across all samples with various expression levels. In HGSOC#4 case, real-time PCR measured nine SVs relative expressions in OM and PELVIS biopsies using breakpoints-spanning primers/probe sets (Figure 3D).

Taken together, a total of 29 SVs breakpoints-spanning tumor-informed primers/probe sets were tested, and only one set showed no detection. The rest of the SVs were detected in the corresponding biopsy sites suggested by the WGS analysis (Figure 2) without detection in matched WBC samples. These results provide strong evidence that the TaqMan probes have high specificity in tumor-informed SVs detection.

### Measurements of SVs concentration in HGSOC multisite biopsies by ddPCR

**Figure 4:**
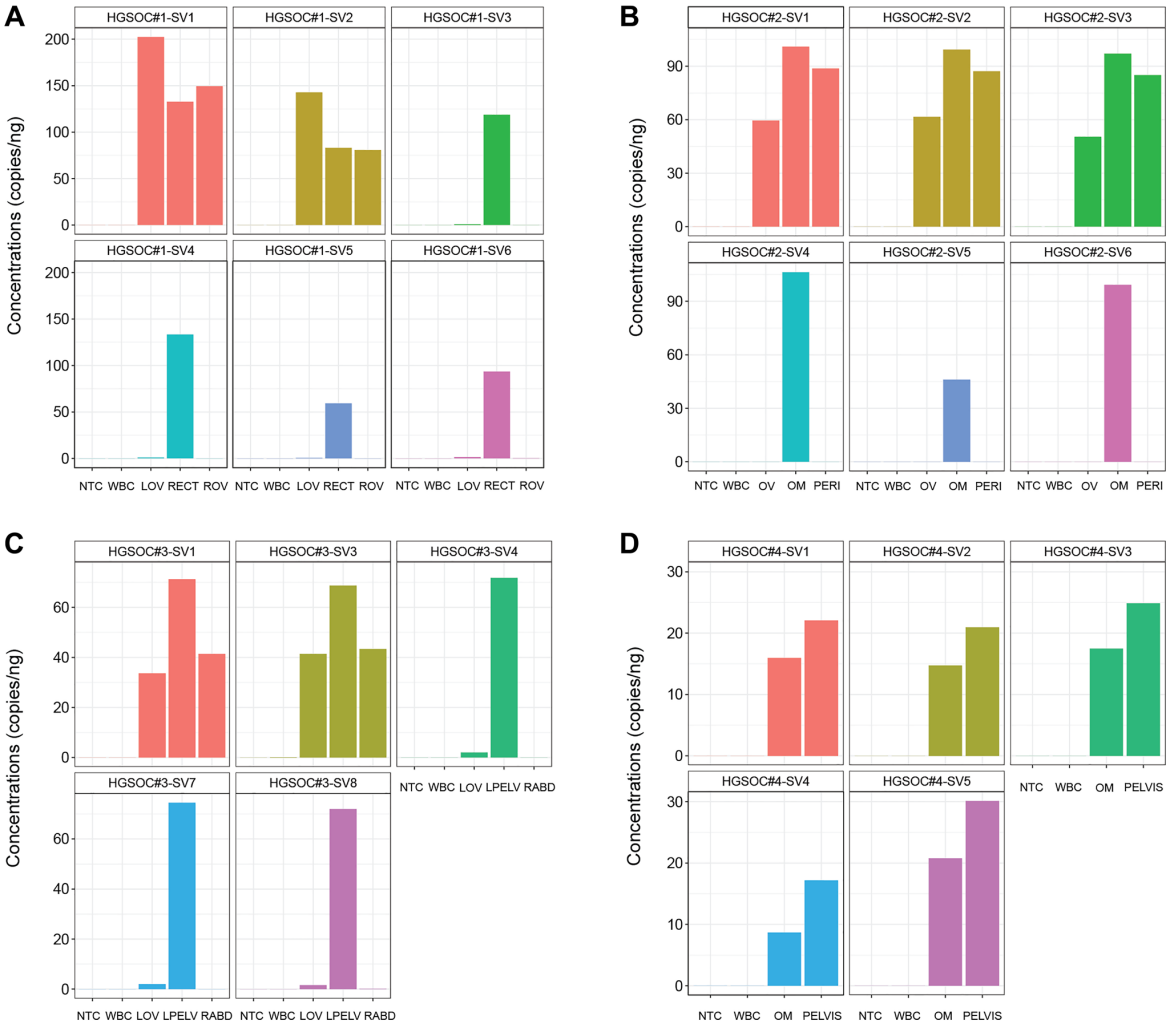
Measurements of SVs concentrations in HGSOC multisite biopsies. (**A**–**D**) Concentrations (copy numbers per ng sample input) of targeted SVs were measured by ddPCR (digital droplet PCR). Each column represents the mean value of three replicates. Germline control WBC of each HGSOC and NTC were used for all experiments. (A) In HGSOC#1, gDNA of multisite biopsies LOV (left ovary), ROV (right ovary), and RECT (rectus) were used ddPCR and 6 SV targets (SV1-6) were measured. (B) In HGSOC#2, gDNA of multisite biopsies OV (ovary), OM (omentum), and PERI (pericardium) were used for ddPCR and 6 SV targets (SV1-6) were measured. (C) In HGSOC#3, gDNA of multisite biopsies LOV(left ovary), LPELV (left pelvis), and RABD (right abdominum) were used for ddPCR and 5 SV targets (SV1, 3, 4, 7, 8) were measured. (D) In HGSOC#4, gDNA of biopsies OM (omentum) and PELVIS (pelvis) were used for ddPCR and 5 SV targets (SV1-5) were measured.

To increase the sensitivity in the final application of SVs measurement in liquid biopsy, we applied ddPCR for selected primers/probe sets in multisite biopsies of all four HGSOCs. In HGSOC#1 and #2, we were able to measure SV1-6 of HGSOC#1, and SV1-6 of HGSOC#2 in their corresponding multisite biopsies (Figure 4A, 4B), including SV5 of HGSOC#1, which was not detected by real-time PCR (Figure 3A). As shown in Figure 4A, in HGSOC#1, SV1 and SV2 were expressed across all biopsies, while the rest SVs were mainly expressed in the RECT site. Similarly, in Figure 4B, in HGSOC#2, SV1, SV2, and SV3 had universal expression, but inHGSOC#2, SV4, SV5, and SV6 specifically expressed in the OM site.

In HGSOC#3 and #4, due to volume limitations of multisite biopsies, we selected five SVs for each case to measure their concentrations by ddPCR. In HGSOC#3, SV1 and SV3 were measured in all biopsy sites, while SV4, SV7, and SV8 were mainly in the LPELV site (Figure 4C). In HGSOC#4, only two biopsies were obtained, and the selected SVs were measured in both sites (Figure 4D).

In summary, ddPCR showed superior sensitivity in measuring concentrations of SVs using the breakpoints-spanning primers/probe sets compared to real-time PCR. Validated ddPCR assays are ready to use in the absolute quantification of tumor-informed SVs in liquid biopsy.

### Quantification of HGSOC tumor-informed SVs from liquid biopsy

After the validation of the breakpoints-spanning primers/probe sets in multisite biopsies by ddPCR, we used selective sets to measure the SVs concentrations from the corresponding cfDNA isolated from fresh frozen plasma. We obtained pre-treatment plasma from all four HOSOC patients. For the HGSOC#3 case, besides the pre-treatment/surgery plasma, we also obtained post-surgery plasma. We requested 11 healthy donors’ fresh frozen plasma from the gynecological oncology tumor bank at MD Anderson. After isolation and quality control of all healthy donors’ cfDNA, we pooled the cfDNA and used it as the healthy donor cfDNA control for this study.

**Figure 5:**
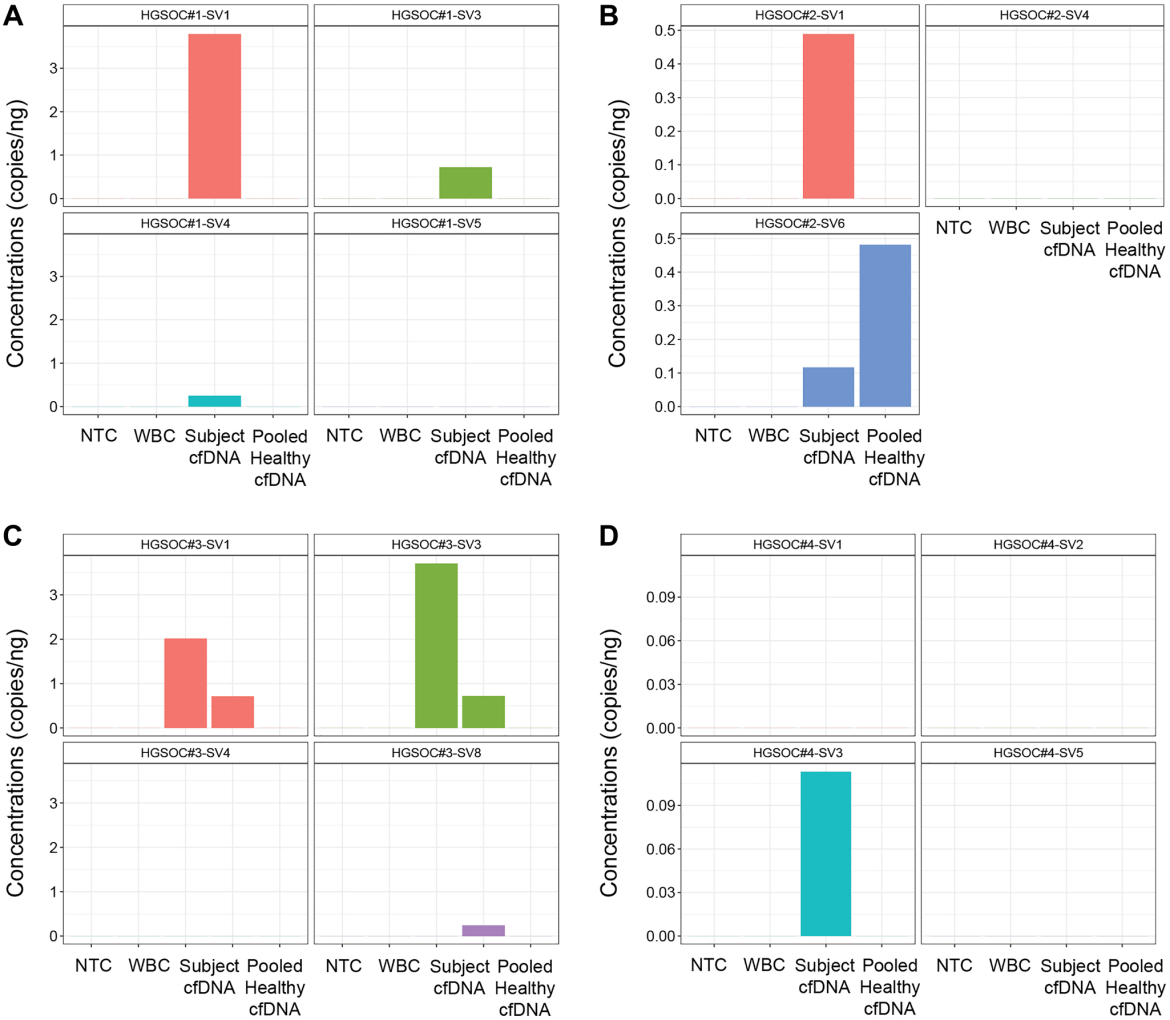
Quantification of tumor-informed SVs of HGSOCs in liquid biopsy. (**A**–**D**) Concentrations (copy numbers per ng sample input) of targeted SVs were measured by ddPCR (digital droplet PCR). Each column represents the mean value of 1 or 2 replicates due to sample limitations. Germline control WBC(white blood cell) and subject pre-treatment cfDNA (cell-free DNA) of each HGSOC, pooled healthy cfDNA and a NTC were used for all experiments. (A) In HGSOC#1, 4 SV targets (SV1, 3, 4, 5) were measured. (B) In HGSOC#2, 3 SV targets (SV1, 4, 6) were measured. (C) In HGSOC#3, subject post-surgery cfDNA was also used in ddPCR and 4 SV targets (SV1, 3, 4, 8) were measured. (D) In HGSOC#4, 4 SV targets (SV1, 2, 3, 5) were measured.

For the HGSOC#1, we selected four SVs to measure their concentrations, three SVs were detected from the pre-treatment liquid biopsy (Figure 5A). For HGSOC#2, three SVs were measured in the liquid biopsy, two had positive measurements in the patient’s liquid biopsy. However, in HGSOC#2, SV6 also had a higher measurement in the healthy donor cfDNA (Figure 5B).

In HGSOC#3, we measured four SVs concentrations in this patient’s pre- and post-surgery cfDNA. SV1 and SV3 had positive measurements in both pre- and post-surgery liquid biopsies, with higher expression in the pre-surgery cfDNA; SV8 was only detected in the post-surgery liquid biopsy (Figure 5C). In HGSOC#4, among the four SVs we measured in the cfDNA, SV3 showed a positive signal (Figure 5D).

These results indicated the possibility of measuring tumor-informed SVs of HGSOCs from liquid biopsy using personalized TaqMan probes by ddPCR.

## DISCUSSION

Previous studies have attempted to utilize cfDNA to monitor solid tumor disease burden, however, few efforts or results have been made in HGSOCs, especially using SVs which is a characteristic feature of this disease. Our results for the first time used multisite biopsies to identify personalized SVs in liquid biopsy without pre-amplification of cfDNA. These results demonstrated the feasibility of detecting tumor-informed SVs from cfDNA, especially the shared-SVs across multisite biopsies. We established a practical workflow with high sensitivity and specificity that may be useful for monitoring disease burden of HGSOCs. One benefit of this workflow is that there is no requirement of healthy donor cfDNA as a control during real-time application, as each primers/probe set is personalized which is unique to a patient of interest, therefore the WBC of the patient will provide sufficient germline control.

Despite the appealing significance of this study, several key limitations should be noted. One limitation is that this pilot study has limited statistical power and generalizability due to a small group of cases studied. One thing makes it challenging for utilization in actual practice is that multisite biopsies with high tumor content work better for this workflow, but these materials are not routinely available in clinical practice. Another limitation is that as a tumor-informed approach, this method has a high cost and significant practical barriers for non-research implementation. Besides the hurdles to overcome before application of the workflow in clinical practice, there are some practical limitations of the designing process: (1) difficulty identifying high confidence SVs from biopsies, mostly because of sample quality like volume, tumor content and necrosis percentage, etc; (2) during the process of primer/probe sets designing and screening, there were approximately 50% screened out due to no amplification in any samples or unspecific amplification in germline control, which makes the screening process sometimes longer than expected. Lastly, we also attempted to identify subclonal SVs with high confidence, however, current published algorithms are limited.

Our study has opened various opportunities for further exploration. Refining the ability to detect and validate subclonal SVs deserves a decent amount of effort and time. Achievement of this task will enable clonal and subclonal SVs labeling and tracking, so that following recurrence, correlate cfDNA trajectories with composition of recurrent tumor. To further expand this study, the workflow should be tested by longitudinal sample collections, for example, comparisons between before and after surgery or chemotherapy. Larger cohort and/or as a translational component of a prospective clinical trial is warranted.

## MATERIALS AND METHODS

### Blood and tumor samples

Pre-treated cryopreserved multisite tumor biopsies, WBCs and plasma from four patients diagnosed with HGSOCs and 15 healthy donor plasmas were obtained from the M.D. Anderson Cancer Center Gynecologic Oncology Multidisciplinary Tumor Bank under an Institutional Review Board (IRB)–approved biospecimen use protocol (#2022-0078). Written, informed consent for biospecimen collection and research use was provided by each patient under IRB protocol (LAB02-0188). Samples inclusion criteria are: cryopreserved, pre-treatment, pathological diagnosis with HGSOC, multisite biopsies (at least 2 sites), and paired plasma with volume at least 1 mL. The biopsies and plasma were all collected at the pre-treatment time point. In the HGSOC#3 only, besides the pre-treatment biopsies and plasma, after-surgery plasma was also collected. Each biopsy specimen was reviewed by an expert gynecologic oncology pathologist (B.L.) and confirmed to contain at least 50% tumor cells with less than 20% necrosis. Approximately 25 mg of each biopsy specimen, 100 μL of WBC and 1 mL of plasma were acquired for sequencing and other analyses.

### Cell lines

Ovarian cancer cell lines Caov-3 [Caov3] (ATCC HTB-75) and SK-OV-3 [SKOV-3; SKOV3] (ATCC HTB-77) were purchased from ATCC. Caov-3 were cultured in Dulbecco’s Modified Eagle’s Medium (DMEM) with high glucose (Cytiva, cat. #SH30285.FS) and SKOV-3 were cultured in McCoy’s 5a Medium Modified (Cytiva, cat. #SH30200.01). Both mediums were supplemented with 10% heat-inactivated FBS (Life Technologies, cat. #16140071) and 1% penicillin/streptomycin (Cytiva, cat. #SV30010). All the cells were grown at 37°C in a humidified incubator with 5% CO_2_.

### Generation of synthetic cfDNA

Genomic DNA of cell lines Caov-3 and SKOV-3 were isolated using the DNeasy Blood and Tissue Kit (Qiagen, cat. #69504) per the manufacturer’s instructions. Genomic DNA was sheared using an ME220 Focused-ultrasonicator (Covaris, Inc.) for 5 minutes under the following setup: base pair mode 150bp, repeat 23, peak power 50W, duty factor 30%, cycles per burst 1000. The size distribution of sheared synthetic cfDNA was confirmed using a TapeStation (Agilent, Inc.; cat. #G2964AA). Concentrations were measured using Qubit^™^ dsDNA HS Assay Kit (Thermo Fisher Scientific, cat. #Q32851) by Qubit^™^ 4 Fluorometer (Thermo Fisher Scientific, cat. #Q33238).

### Genomic DNA isolation

Genomic DNA from multisite biopsy specimens was isolated using the DNeasy Blood and Tissue Kit (Qiagen, cat. #69504) per the manufacturer’s instructions. Concentrations were measured using Qubit^™^ dsDNA BR Assay Kit (Thermo Fisher Scientific, cat. #Q32850) by Qubit^™^ 4 Fluorometer (Thermo Fisher Scientific, cat. #Q33238). Genomic DNA purity was analyzed by NanoDrop 2000 spectrophotometer (ThermoFisher Scientific, cat. #ND-2000).

### Whole genome sequencing (WGS)

WGS of tumor and normal samples was performed by the MD Anderson Cancer Center Cancer Genomics Laboratory similar to how has been previously described [14] with target depth of 60X for tumor samples and 30X for normal controls. Briefly, 500 ng of genomic DNA was used for library preparation using bead-based tagmentation. A dual-indexed paired-end read of 320 cycle run is conducted to complete the sequencing process on a NovaSeq 6000 system (Illumina, Inc.).

### Structural variants calling

Sequencing reads were aligned to the GRCh38 reference genome using BWA [15]. Somatic SV callers used in cancer research often show limited concordance, and combining multiple tools is recommended to reduce false positives [16]. For multi-site tumor samples with their matched normal blood control, we applied five somatic SV detection tools, including GRIDSS 2.13.2 [17], DELLY 1.16 [18], SvABA 1.2.0 [19], Manta 1.6 [20], and BRASS v6.2.1 [21], using default parameters. The detected SVs were first reformatted using AnnotSV 3.4.1 [22], and merged using the SURVIVOR [23] merge function, considering breakpoints located within 1,000 base pairs of each other as the same event. In HGSOC#1–#3, SVs detected by all five tools were considered as high-confidence SVs. In HGSOC#4, due to reduced SV detection associated with low tumor purity, the threshold was relaxed, and SVs supported by at least four of the five were considered as high-confidence.

### cfDNA isolation and quality control

cfDNA was isolated using QIAamp Circulating Nucleic Acid Kit (Qiagen, cat.#5514) per the manufacturer’s instructions. cfDNA was analyzed using cell-free DNA ScreenTape (Agilent, cat. #5067-5630) and reagents (Agilent, cat. #5067-5631) by Agilent 2200 TapeStation system (cat. #G2964AA). Concentrations were measured using Qubit^™^ dsDNA HS Assay Kit (Thermo Fisher Scientific, cat. #Q32851) by Qubit^™^ 4 Fluorometer (Thermo Fisher Scientific, cat. #Q33238).

### SV Breakpoints-spanning primers design

Coordinates sequences around the breakpoint 1 (−150bp ~ +10bp) and breakpoint 2 (−10bp ~ +150bp) of SVs were acquired from UCSC Genome Browser using Genome Reference Consortium Human GRCh38.p14 (GCA_000001405.29). The combined coordinate sequence of breakpoint 1 and 2 were used as a template for primers design by Primer3 [24]. Upstream primer located in breakpoint 1 coordinate and downstream primer located in breakpoint 2 coordinate were selected to ensure the amplicon spanning across both breakpoints of SV. Primers were tested by PCR using AmpliTaq Gold 360 Master Mix (Thermo Fisher Scientific, cat. #4398881). PCR amplicon was subjected to agarose gel electrophoresis for analysis.

### Custom TaqMan Probe design

PCR amplicon for each SV was cloned using TOPO TA Cloning Kit for Sequencing (Thermo Fisher Scientific, cat. #450030) and transformed into chemically competent bacteria (Thermo Fisher Scientific, cat. #C404010). Plasmids were isolated by QIAprep Spin Miniprep Kit (Qiagen, cat. #27104). Sanger sequencing was performed by the ATGC (The Advanced Technology Genomics Core) facility at UT MD Anderson to obtain the accurate coordinate sequences spanning breakpoints of SVs. Custom TaqMan probes (Thermo Fisher Scientific, cat. #4331348) were designed using the accurate coordinate sequences as templates.

### Real-time PCR

Genomic DNA isolated from multisite biopsy specimens was used as templates for real-time PCR. SV breakpoints-spanning primers/custom TaqMan probes and TaqMan Fast Advanced Master Mix (Thermo Fisher Scientific, cat. #4444557) were utilized. The real-time PCR was performed by using QuantStudio 6 Pro (Applied Biosystems, Inc.) in a 96-well format. The relative concentration (ΔCq) of each SV was used for comparison, and it was calculated as follows: ΔCq = Cq(SV) - Cq(GAPDH).

### Digital droplet PCR (ddPCR)

ddPCR Supermix for Probes (No dUTP) (Bio-rad, cat. #1863024) was used for ddPCR reaction. QX200 AutoDG Droplet Generator (Bio-rad, cat. #1864101) was used for droplet generation. Regular PCR was performed in droplets. QX200 Droplet Reader (Bio-rad, cat. #1864003) was used for droplet reading. SVs concentrations were calculated to copies per ng DNA input. QuantaSoft Software (Bio-rad, Regulatory Edition #1864011) was used for analysis.

## CONCLUSIONS

Chemotherapy is effective at inducing remission for most patients with HGSOC, yet relapse is frequent. Methods to interrogate characteristic molecular properties and disease burden are needed to better understand the mechanisms of HGSOC relapse. To address this unmet need, we developed and validated a novel cfDNA-based approach for tracking on-treatment clonal evolution using tumor-informed SVs. Our results demonstrate the feasibility of detecting tumor-informed SVs from pre-treatment cfDNA using breakpoint-spanning ddPCR and may enable a novel and sensitive method for monitoring on-treatment disease burden.

## SUPPLEMENTARY MATERIALS



## Abbreviations

HGSOC: High grade serous ovarian cancer
cfDNA: cell-free DNA
SV: structural-variant
ddPCR: digital droplet PCR
MRD: minimal residual disease
SNVs: single nucleotides variants
IRB: Institutional Review Board
DMEM: Dulbecco’s Modified Eagle’s Medium
WGS: Whole genome sequencing
ATGC: The Advanced Technology Genomics Core
NTC: no template control reaction
DEL: deletion
EGA: European Genome-phenome Archive

## References

[1] Eisenhauer EL, Salani R, Copeland LJ. 11 - Epithelial Ovarian Cancer. In: DiSaia PJ, Creasman WT, Mannel RS, McMeekin DS, (Eds.). Clinical Gynecologic Oncology. Elsevier; 2018; 253–89.e14. 10.1016/B978-0-323-40067-1.00011-5.

[2] Beltrame L, Di Marino M, Fruscio R, Calura E, Chapman B, Clivio L, Sina F, Mele C, Iatropoulos P, Grassi T, Fotia V, Romualdi C, Martini P, et al. Profiling cancer gene mutations in longitudinal epithelial ovarian cancer biopsies by targeted next-generation sequencing: a retrospective study. Ann Oncol. 2015; 26:1363–71. 10.1093/annonc/mdv164. 25846551

[3] Lambrechts S, Smeets D, Moisse M, Braicu EI, Vanderstichele A, Zhao H, Van Nieuwenhuysen E, Berns E, Sehouli J, Zeillinger R, Darb-Esfahani S, Cacsire Castillo-Tong D, Lambrechts D, Vergote I. Genetic heterogeneity after first-line chemotherapy in high-grade serous ovarian cancer. Eur J Cancer. 2016; 53:51–64. 10.1016/j.ejca.2015.11.001. 26693899

[4] Kwapisz D. The first liquid biopsy test approved. Is it a new era of mutation testing for non-small cell lung cancer? Ann Transl Med. 2017; 5:46. 10.21037/atm.2017.01.32. 28251125 PMC5326656

[5] Hou JY, Chapman JS, Kalashnikova E, Pierson W, Smith-McCune K, Pineda G, Vattakalam RM, Ross A, Mills M, Suarez CJ, Davis T, Edwards R, Boisen M, et al. Circulating tumor DNA monitoring for early recurrence detection in epithelial ovarian cancer. Gynecol Oncol. 2022; 167:334–41. 10.1016/j.ygyno.2022.09.004. 36117009

[6] Beaver JA, Jelovac D, Balukrishna S, Cochran R, Croessmann S, Zabransky DJ, Wong HY, Toro PV, Cidado J, Blair BG, Chu D, Burns T, Higgins MJ, et al. Detection of cancer DNA in plasma of patients with early-stage breast cancer. Clin Cancer Res. 2014; 20:2643–50. 10.1158/1078-0432.CCR-13-2933. 24504125 PMC4024333

[7] Zhang EW, Dagogo-Jack I, Kuo A, Rooney MM, Shaw AT, Digumarthy SR. Association between circulating tumor DNA burden and disease burden in patients with ALK-positive lung cancer. Cancer. 2020; 126:4473–84. 10.1002/cncr.33118. 32757294

[8] Swisher EM, Wollan M, Mahtani SM, Willner JB, Garcia R, Goff BA, King MC. Tumor-specific p53 sequences in blood and peritoneal fluid of women with epithelial ovarian cancer. Am J Obstet Gynecol. 2005; 193:662–67. 10.1016/j.ajog.2005.01.054. 16150257

[9] Parkinson CA, Gale D, Piskorz AM, Biggs H, Hodgkin C, Addley H, Freeman S, Moyle P, Sala E, Sayal K, Hosking K, Gounaris I, Jimenez-Linan M, et al. Exploratory Analysis of TP53 Mutations in Circulating Tumour DNA as Biomarkers of Treatment Response for Patients with Relapsed High-Grade Serous Ovarian Carcinoma: A Retrospective Study. PLoS Med. 2016; 13:e1002198. 10.1371/journal.pmed.1002198. 27997533 PMC5172526

[10] Strickler JH, Loree JM, Ahronian LG, Parikh AR, Niedzwiecki D, Pereira AAL, McKinney M, Korn WM, Atreya CE, Banks KC, Nagy RJ, Meric-Bernstam F, Lanman RB, et al. Genomic Landscape of Cell-Free DNA in Patients with Colorectal Cancer. Cancer Discov. 2018; 8:164–73. 10.1158/2159-8290.CD-17-1009. 29196463 PMC5809260

[11] Patch AM, Christie EL, Etemadmoghadam D, Garsed DW, George J, Fereday S, Nones K, Cowin P, Alsop K, Bailey PJ, Kassahn KS, Newell F, Quinn MC, et al, and Australian Ovarian Cancer Study Group. Whole-genome characterization of chemoresistant ovarian cancer. Nature. 2015; 521:489–94. 10.1038/nature14410. 26017449

[12] Layer RM, Chiang C, Quinlan AR, Hall IM. LUMPY: a probabilistic framework for structural variant discovery. Genome Biol. 2014; 15:R84. 10.1186/gb-2014-15-6-r84. 24970577 PMC4197822

[13] Nik-Zainal S, Davies H, Staaf J, Ramakrishna M, Glodzik D, Zou X, Martincorena I, Alexandrov LB, Martin S, Wedge DC, Loo PV, Ju YS, Smid M, et al. Landscape of somatic mutations in 560 breast cancer whole-genome sequences. Nature. 2016; 534:47–54. 10.1038/nature17676. 27135926 PMC4910866

[14] Hillman RT, Celestino J, Terranova C, Beird HC, Gumbs C, Little L, Nguyen T, Thornton R, Tippen S, Zhang J, Lu KH, Gershenson DM, Rai K, et al. KMT2D/MLL2 inactivation is associated with recurrence in adult-type granulosa cell tumors of the ovary. Nat Commun. 2018; 9:2496. 10.1038/s41467-018-04950-x. 29950560 PMC6021426

[15] Li H, Durbin R. Fast and accurate short read alignment with Burrows-Wheeler transform. Bioinformatics. 2009; 25:1754–60. 10.1093/bioinformatics/btp324. 19451168 PMC2705234

[16] van Belzen IAE, Schönhuth A, Kemmeren P, Hehir-Kwa JY. Structural variant detection in cancer genomes: computational challenges and perspectives for precision oncology. NPJ Precis Oncol. 2021; 5:15. 10.1038/s41698-021-00155-6. 33654267 PMC7925608

[17] Cameron DL, Schröder J, Penington JS, Do H, Molania R, Dobrovic A, Speed TP, Papenfuss AT. GRIDSS: sensitive and specific genomic rearrangement detection using positional de Bruijn graph assembly. Genome Res. 2017; 27:2050–60. 10.1101/gr.222109.117. 29097403 PMC5741059

[18] Rausch T, Zichner T, Schlattl A, Stütz AM, Benes V, Korbel JO. DELLY: structural variant discovery by integrated paired-end and split-read analysis. Bioinformatics. 2012; 28:i333–39. 10.1093/bioinformatics/bts378. 22962449 PMC3436805

[19] Wala JA, Bandopadhayay P, Greenwald NF, O’Rourke R, Sharpe T, Stewart C, Schumacher S, Li Y, Weischenfeldt J, Yao X, Nusbaum C, Campbell P, Getz G, et al. SvABA: genome-wide detection of structural variants and indels by local assembly. Genome Res. 2018; 28:581–91. 10.1101/gr.221028.117. 29535149 PMC5880247

[20] Chen X, Schulz-Trieglaff O, Shaw R, Barnes B, Schlesinger F, Källberg M, Cox AJ, Kruglyak S, Saunders CT. Manta: rapid detection of structural variants and indels for germline and cancer sequencing applications. Bioinformatics. 2016; 32:1220–22. 10.1093/bioinformatics/btv710. 26647377

[21] BRASS: Breakpoints via Assembly - Identifies Breaks and Attempts to Assemble Rearrangements in Whole Genome Sequencing Data, and Github.

[22] Geoffroy V, Herenger Y, Kress A, Stoetzel C, Piton A, Dollfus H, Muller J. AnnotSV: an integrated tool for structural variations annotation. Bioinformatics. 2018; 34:3572–74. 10.1093/bioinformatics/bty304. 29669011

[23] Jeffares DC, Jolly C, Hoti M, Speed D, Shaw L, Rallis C, Balloux F, Dessimoz C, Bähler J, Sedlazeck FJ. Transient structural variations have strong effects on quantitative traits and reproductive isolation in fission yeast. Nat Commun. 2017; 8:14061. 10.1038/ncomms14061. 28117401 PMC5286201

[24] Untergasser A, Cutcutache I, Koressaar T, Ye J, Faircloth BC, Remm M, Rozen SG. Primer3--new capabilities and interfaces. Nucleic Acids Res. 2012; 40:e115. 10.1093/nar/gks596. 22730293 PMC3424584

