# Supplementary material for: Tumor-informed liquid biopsy detection of structural variants in high grade serous ovarian cancer
Source: Oncoscience. 2026 Mar 5;13:44–54. doi: 10.18632/oncoscience.645 (PMC12981705; doi:10.18632/oncoscience.645)
Supplement: Supplementary file 1 [file oncoscience-13-645-s001.pdf]

Tumor-informed liquid biopsy detection of structural variants in high grade serous ovarian cancer

SUPPLEMENTARY MATERIALS

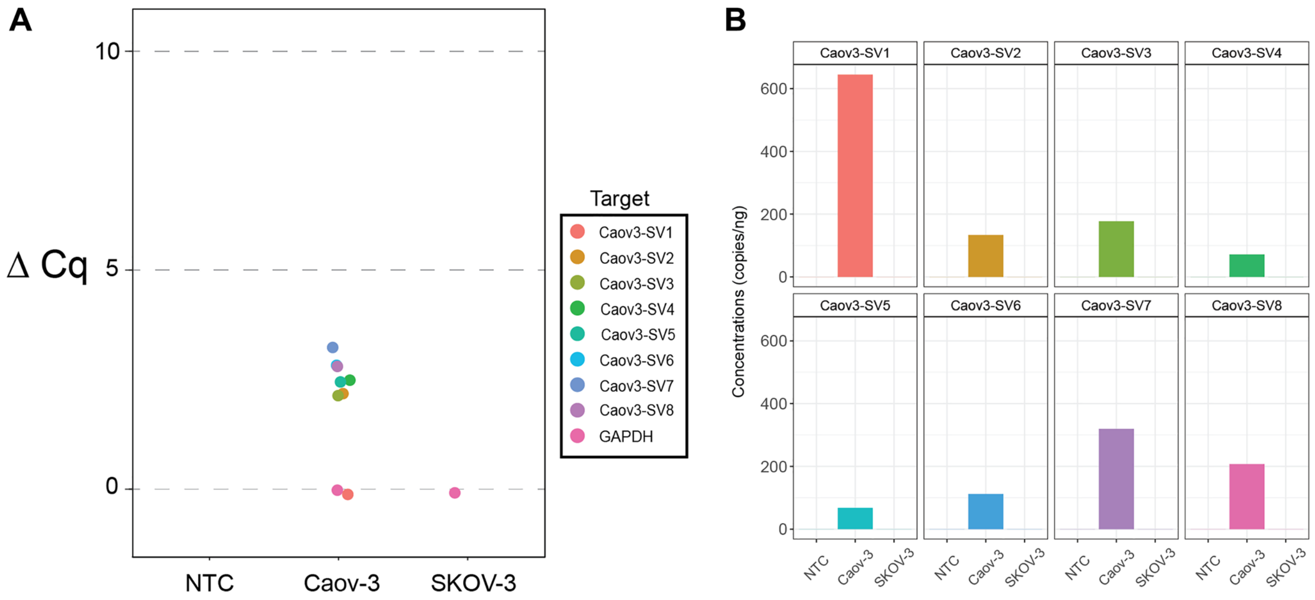

Supplementary Figure 1: Optimization of workflow by ovarian cancer cell lines. (**A**) Real-time PCR results of targeted Caov-3 SVs. ΔCq (ΔCq = Cq.Target - Cq.GAPDH) were calculated and compared in synthetic cfDNA of Caov-3 and SKOV-3 (serving as a control). Each dot represents the mean value of three duplicates. NTC (no template control) was used as a control. 8 Caov-3 SVs were tested. (**B**) Concentrations (copy numbers per ng sample input) of targeted Caov-3 SVs were measured by ddPCR (digital droplet PCR). Each column represents the mean value of three replicates. The same targets and samples were used as in Supplementary Figure 1A.

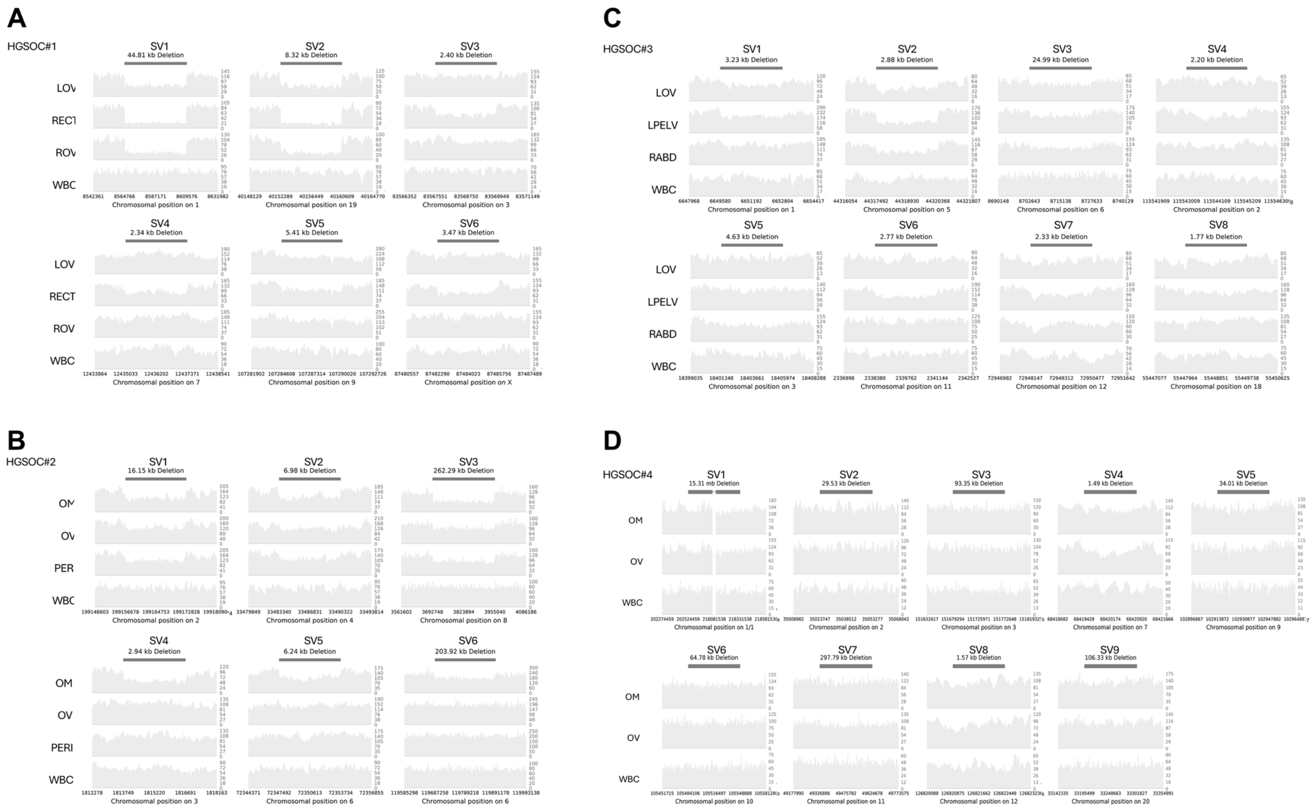

Supplementary Figure 2: Visualization of large deletions using Samplots. Sequence coverage across selected large deletions (SV) in HGSOC #1-#4 cases is shown for each sampling site (OM, OV, PERI, LOV, RECT, ROV, LPELV, RABD) and white blood cells (WBC). The Y-axis indicates coverage depth. Horizontal grey bars mark the deletion size. (**A**) HGSOC#1: Coverage for SV1–SV6 across four tissues (LOV, RECT, ROV, WBC). (**B**) HGSOC#2: Coverage for SV1–SV6 across four tissues (OM, OV, PERI, WBC). (**C**) HGSOC#3: Coverage for SV1–SV8 across four tissues (LOV, LPELV, RABD, WBC). (**D**) HGSOC#4: Coverage for SV1–SV9 across three tissues (OM, OV, WBC).
